# Self-Reported Use and Reasons among the General Population for Using Sports Nutrition Products and Dietary Supplements

**DOI:** 10.3390/sports4020033

**Published:** 2016-06-07

**Authors:** Floris Wardenaar, Remko van den Dool, Ingrid Ceelen, Renger Witkamp, Marco Mensink

**Affiliations:** 1 HAN University of Applied Sciences, Nijmegen 6525 AJ, The Netherlands; Ingrid.ceelen@han.nl; 2 *Mulier Instituut*, Utrecht 3584 AA, The Netherlands; r.vandendool@mulier.nl; 3 Wageningen University, Wageningen 6700 EV, The Netherlands; Renger.witkamp@han.nl (R.W.); Marco.mensink@wur.nl (M.M.)

**Keywords:** nutritional supplements, sports dietetics, exercise, health, performance

## Abstract

The purpose of the present study was to determine the prevalence of dietary supplements (DS’s) and sport nutrition product (SNPs) among the general population, to identify differences for gender, age, and exercise frequency, and to determine the main reasons for use. The study was designed as a web-based questionnaire in a representative sample (*n* = 1544) of the Dutch population. Sixty-two percent (*n* = 957) of the respondents reported having used DS’s, SNPs, or both in the last twelve months. Women and older people reported the highest DS use. The highest use of SNPs was reported by regular exercising men and younger people with improving sporting performance as their main objective. Most frequently reported DS’s were multivitamins (28%) and vitamin C (19%)—for SNPs, energy drinks (22%) and isotonic drinks (19%). Health considerations were the most important motivation (DS’s 90% and SNPs 52%), but also performance was substantially reported (DS’s 14% and SNPs 35%). A substantial group of sedentary respondents also reported the use of SNPs. This study confirms that DS’s, SNPs, or both are widely used among the general population. Both health as performance are important reasons for use. It can be questioned whether the use of SNPs fits all respondents’ physical activity needs.

## 1. Introduction

Nutritional supplements such as dietary supplements (DS’s) and sports nutrition products (SNPs) are commonly used by athletes to maximize their performance, to accelerate recovery, to prevent nutrient deficiencies, and to maintain good general health [1,2,3,4]. However, these arguments give the impression that they are also often used by physically less active people and that these products are actively promoted by suppliers aiming to reach a wider target group [5,6].

The majority of the literature investigating DS and SNP use does combine both as nutritional supplements or only focuses on dietary supplements, despite the fact that SNPs do not only contain macronutrients but also micronutrients. Moreover, SNPs are increasingly found on the shelves of supermarkets, making these types of products easily accessible for the general population. Thus, from a nutritional perspective, it is necessary to include SNPs in dietary analysis.

Consumers of both DS’s and SNPs may use these preparations with the intention of maintaining good health [5], often unaware of the supporting scientific evidence—or lack thereof [6]. Further, it can be questioned whether they also use these type of products for performance reasons. Surveys on dietary supplement consumption by the general population generally focus on the use of multivitamins or other micro-nutrient products, but often make no distinction between sport-specific DS’s and SNPs, or make no mention of them at all [7]. Until now, this has only been investigated in small groups of non-athletes included as controls to compare their use with the nutritional supplement use of elite athletes [1,6,8]. However, two of these three studies did not provide a clear insight into the contribution of sport-specific SNPs used in the non-athletes group [1,8]; therefore, obtaining additional information in a large sample of a general population can be seen as valuable.

From an earlier pilot survey among general consumers in the Netherlands, we concluded that a typical user of sports supplements and sports nutrition products is a young (<35 years) male, who regularly takes part in sports (>once/week) and is a member of a sports club [5]. When subjects were asked about their reasons for using DS’s and SNPs, we found a lower prevalence of sports-oriented use (5.8% for DS’s and 21.6% for SNPs) compared to other studies [6,8].

Therefore, as part of the Dutch National Sport Study [9], we investigated the self-reported use and the main reasons for use of DS’s and SNPs. The objectives of this survey were (a) to determine the prevalence in the Dutch general population of dietary supplement and sports nutrition product use, in general and for specific DS’s and SNPs; (b) to identify differences in use for gender, age, and exercise frequency; and (c) to determine the main reasons for using DS’s, SNPs, or both, particularly for physical performance purposes in comparison to general health purposes. Knowledge of the prevalence and motivation for use of DS’s and SNPs, especially in non-athletes, is of relevance for (sports) dieticians and health professionals giving dietary advice.

## 2. Materials and Methods

### 2.1. Study Design

The present study was part of the Dutch Sport nutrition and Supplement Study (DSSS), which had been approved by the Ethics Board of Wageningen University. To obtain insight in nutritional supplement use of a representative sample of the general population questions were integrated in the Dutch National Sport Study (NSO). GfK Panel Services Benelux BV (GfK) conducted the survey between 22 November 22 and 4 December 2013 using an Internet-based questionnaire (Bellview, Pulse Train, North Palm Beach, FL, USA). To reduce selection bias, the questionnaire was given a neutral title: “Health and exercise”. Moreover, those respondents submitting a completed questionnaire were rewarded with an incentive (bonus points with a value of €1.60 that could be spent within the GfK panel rewarding system), which encouraged those respondents that were not primarily interested in health and exercise to participate. The average duration for filling out the total NSO questionnaire was 16 min, with 2 min for the sport-specific nutrition questions.

### 2.2. Study Population

Out of a panel of 10,000 representative households, 2350 participants received an invitation to fill out the questionnaire. Within thirteen days, 1544 questionnaires were returned (response percentage of 66%), which was considered to deliver a representative sample of the population in the Netherlands for example in terms of age (15–80 years), gender (50%–50% men and women), education level (35% lower education; 42% middle-education; 23% higher education), and ethnicity (80% autochthonous; 8% Western-immigrant; 12% non-Western immigrant).

### 2.3. Questions

To investigate the prevalence of dietary supplement and sports nutrition product use, in general and for specific DS and SNP subcategories, a questionnaire was developed that examined the self-reported use of DS’s and SNPs in the previous 12 months. All products questioned are shown in Table 1. Dietary supplements were products classified as supplements that prevent or treat a perceived nutrient deficiency, *i.e.*, vitamins, minerals, and essential fatty acids. Ergogenic supplements were also included in this category, because they could be used for health purposes as well as performance improvement. Sport nutrition products were macronutrient containing beverages, *i.e.*, sports drinks (electrolyte- and carbohydrate-containing drinks for rehydration and refuelling and energy drinks containing mainly carbohydrate for refuelling) and recovery drinks (containing a combination of carbohydrate and protein aiming to optimize post-exercise recovery), to provide a more convenient form of nutrients in situations where everyday foods are not practical—particularly to address the nutritional needs/goals of an exercise session. All DS’s that could be selected were pre-defined, with an option to indicate “none” or “other” use; in case of SNPs, the option “none of these supplements” was included.

The questions asked were: (1) Which dietary supplements have you used in the last twelve months? (pre-selected options); (2) Which sports nutrition products have you used in the last twelve months? (pre-selected options); (3) and (4) Please indicate the main reason for using these products (DS’s and(or) SNPs). Subjects were asked to select only one option: for health purposes, for physical performance purposes, to improve both, or for other purposes. Gender and sporting frequency were detected as part of the general questionnaire. Respondents were asked to select one or more sports out of a pre-specified list of 44 sports and an “other” option. The exercise frequency (per year) was obtained by an open question that was part of the annual NSO questionnaire: How many times have you exercised in the last twelve months?

### 2.4. Statistical Analysis

After terminating the registration period results were entered into SPSS (IBM SPSS Statistics, version 20, IBM Corporation, New York, United States). All self-reported results are given as percentages. For each value, a 95% confidence interval was estimated and expressed as a percentage based on the following formula √(p(1-p)/n), of which p is the sample proportion and n is the sample size. Individual (2 × 2) chi-square tests were used to assess whether there was a difference in the expected distribution of DS and SNP use within tied subcategories such as gender, age, and sporting frequency. Results were considered significantly different at *P* ≤ 0.05. No correction was calculated for multiple comparisons because of the relatively small number of equations between each subcategory.

## 3. Results

A response rate of 66% within 13 days resulted in a sample size of *n* = 1544 with comparable numbers of men (*n* = 791) and women (*n* = 753). The largest proportion, 76% of the sample, was aged between 21 and 65 years. Of the total study population, 35% reported that they were not engaged in any sporting activity or only 1–11 times a year in the last twelve months and are considered sedentary, while 31% of the population exercised between 12 and 59 times a year. One third of the study population (33%) indicated that they were a member of a sports club, and most of the subjects performed recreational sports without a competitive element (77%). Table 1 contains numbers (n) of participants in specific subgroups.

### 3.1. Prevalence

The most reported DS’s were multivitamin/mineral supplements, vitamin C, and vitamin D. The most frequently reported SNPs were energy drinks and isotonic drinks (Table 1). With regard to total prevalence of DS and SNP use in total, 62% (95% CI: 60–64) of the study population reported the use of DS’s, SNPs, or both. The percentage use of products categorized as DS’s or SNPs was only 28% (95% CI: 26–30) and 12% (95% CI: 10–14), respectively. In total, 38% (95% CI: 36–40) did not report using any type of product (Table 2).

As shown in Table 1, men reported using more SNPs, such as energy drinks, isotonic drinks, and recovery drinks, than women, except for protein shakes and energy gels. Women reported a higher proportion of vitamin and mineral supplements, essential fatty acids, probiotics, and other DS’s than did men. No differences between men and women were seen for the use of ergogenic supplements, such as caffeine and creatine.

The use of dietary supplements was different between age categories, as can be seen in Table 1. For example, vitamin D and calcium supplement use was higher in the oldest age category of 66–80 years. Moreover, magnesium was high in both the youngest age group of 15–20 years and the oldest age groups of 51–65 and 66–80 years. The sport-specific use of caffeine (defined as tablet/capsule, energy drink/sport nutrition product, coffee, or an equivalent) was especially high in the two youngest age groups of 15–20 and 21–35 years old. Regarding SNPs, energy drink, isotonic drink, and protein shake prevalence was the highest in the two youngest age categories of 15–20 and 21–35 years. The prevalence of the use of recovery drinks declined as age increased. In general, some differences between age groups existed, resulting in a higher prevalence in the use of SNPs or in the combination of DS’s and SNPs (*i.e.*, using both) in the younger categories (15–20 years and 21–35 years) compared with the older age categories (51–65 years and 66–80 years), as shown in Table 2.

The part of the population that exercised >60 times a year reported the greatest consumption of SNPs. This group also reported the highest percentages for the single product use of multivitamin and minerals, vitamin C, isotonic drinks, energy drinks, protein drinks, recovery drinks, and energy gels (Table 1). People who did not exercise, or who exercised less than 11 times a year, reported the highest non-use (51%) of DS’s and SNPs (Table 2). This percentage was significantly higher than all categories with active participation in sports. Nevertheless, of those participants reporting no exercise, a relatively large number still indicated using SNPs (8%, (95% CI: 5–11)) or a combination of DS’s and SNPs (7%, (95% CI: 5–9)). The frequency of SNP use found in those not exercising was equal to those exercising 1–11 times a year in the case of those reporting only the use of SNPs (8% (95% CI: 5–11) *vs.* 9% (95% CI: 4–14)). This was not the case for combined use of DS’s and SNPs between those not active and those exercising 1–11 times a year (7% (95% CI: 5–9) *vs.* 24% (95% CI: 17–31)). Overall, this resulted in a relative large group of sedentary individuals using SNPs.

### 3.2. Reasons

Most subjects reported health reasons as the main motivation for using DS’s, with a range of 61–89% among categories (Table 3). Improvement of physical performance was not frequently selected as a single reason for use (range 1–8% for different subcategories), although the combined reason (health *and* performance) was reported frequently in some but not all groups. This was most frequently seen in the age group of 15–20 years (25% (95% CI: 19–31)), and the group exercising >120 times a year (22% (95% CI: 18–26)). Furthermore, for SNP use, health reasons were more frequently indicated than performance reasons, with, for health, a range of 17–43% among categories (Table 3), although “other reasons” was answered more frequently (range: 24%–67%). The group exercising >120 times, in comparison with other subcategories, most frequently reported “physical performance purposes” as reasons for use: 23% (95% CI: 19–27] indicated performance as the sole reason, while 34% (95 CI: 29–39) indicated the combined reason of health and performance as the main argument for use.

Women more frequently reported health reasons as their motivation for DS use than men (84% (95% CI: 80–88) *vs.* 72% (95% CI: 69–75)), but not for SNPs (33% (95% CI: 31–35) *vs.* 25% (95% CI: 23–27)). Men reported higher percentages for physical performance purposes and a combination of health and physical performance purposes as the most important reason for DS and SNP use than women.

## 4. Discussion

Almost two-thirds of the respondents indicated having used DS’s, SNPS, or both in the previous twelve months. DS’s were used more frequently than SNPs, with improving health being the most important motivating factor. The most frequently reported products used were, for DS’s, vitamin and mineral supplements, and, for SNPs, energy drinks. DS use was most frequently reported by women and older people. On the other hand, men, young people, and those exercising most frequently reported the highest use of SNPs. Women tended to indicate general health considerations more frequently compared to men as their main reason, whereas men reported physical performance purposes as their main reason for use more often than women. Remarkably, even sedentary respondents reported a relatively high prevalence of SNP use.

### 4.1. Prevalence and Reasons

Combined use of DS and SNP intake of specific groups and populations has been previously reported, ranging from 22% to 98% based on different methods [1,2,3,4,6,8,10,11,12,13,14,15,16,17,18], while we measured a substantial percentage of 61% (combining both DS’s and SNPs) in the current study. Only a few studies have focused on the sport-specific use of DS’s and SNPs in non-athletes [1,6,14]. Although the combined DS and SNP use in the present study is lower than in most recent studies with athletes [2,3,4], it is much higher than the reported 4.8% in a small group of non-athletic controls (*n* = 60) as presented previously [1]. This may be explained by geographical differences indicating the relevance of this type of study for specific regions worldwide.

Overall DS use in this Dutch sample (49%) is similar to DS use of a representative sample of the US population (*i.e.*, 52% [19]) and data based on the latest Dutch Food Consumption Survey (VCP) [20]. This VCP was also questioning dietary supplement use throughout the year, and showed a range of 26–36% for men and 34–54% for women across different age groups. The results of the present study are at the upper end of the results of the VCP, most likely due to including or not including specific types of DS’s (for example ergogenic supplements), but also reflecting some of the differences in our population with those of the VCP (maybe as a result of including those interested in a questionnaire about health and exercise), despite the fact that we included a representative sample of the Dutch population. As observed previously, multivitamins, vitamin C, and vitamin D were most frequently reported [19,20,21].

Gender differences found in the present study are in line with results found earlier in a study including 1689 non-athletes as controls of an athletic population [8]. These authors reported a difference in total nutritional supplement use—a combination of DS’s and SNPs—between men (32%) and women (52%) [8]. We found a similar gender difference although the total prevalence of DS and SNP use was higher in our study, and the difference between both groups was much smaller: 60% *vs.* 64% for men and women, respectively. Interestingly, the women in our study reported higher DS use compared with men (36% *vs.* 20%), while men reported a higher prevalence of SNPs. This gender difference was also previously detected by others [2,8,13,22]. An explanation might be that DS’s are more frequently linked to health, whereas SNPs are more frequently associated with physical performance. This would be in line with our observations that women reported “health” more often as the reason for use, while men reported “physical performance.” In contrast, some studies did not find a gender difference [3] or in fact found the opposite [1,12], which may also depend on the definitions used for DS’s and SNPs.

It is interesting to note that, compared with older respondents, younger respondents use more SNPs or both SNPs and DS’s. This contrasts with some previous investigations that indicated a higher prevalence of nutritional supplement use in adult athletes compared with adolescents [23,24]. It can be speculated that SNPs, such as sports drinks and energy drinks, have become increasingly popular among younger individuals in recent years. This might be attributed to marketing strategies of companies producing these type of products that are appealing to young athletes, resulting, for example, in parents buying these drinks at the supermarket for their children to replenish energy and fluid at half-time during their sports activities on the weekend.

Our findings are also in line with the National Health and Nutrition Examination Study. That study revealed comparable trends for those being active (>120 times a year in the present study *vs.* vigorous in NHANES), reporting the highest percentages for total DS use, but also for the top ranked products like multivitamin/mineral and vitamin C [19].

### 4.2. Strengths and Limitations

The novelty of this paper is that we examined the use of DS’s and SNPs in a representative sample of a general population in relation to reasons for use, *i.e.*, health purposes, physical performance purposes, both, or other reasons, for the total category of DS’s, SNPs, or combined use of DS’s and SNPs. To the best of our knowledge, this is the first study investigating the general reason for use (health, performance, or both) of both DS’s and SNPs in a general population. Besides this, we used a sound sampling strategy with a strong response rate.

An important added value of the present study is that it evaluated the prevalence of intake of DS’s and SNPs separately, as well as the different types of products used—an insight which has generally not been provided in other large cohort publications [19,20,21,25,26]. For example, there does not seem to be any nutritional substantiation for energy-containing SNPs used by the majority of the non-exercising population. Combining DS’s and SNPs will not provide this insight.

The estimated proportions with a confidence interval (CI) of 95% are relatively small, which increases the likelihood that the true value of the respondents will actually lay close to the reported average percentage.

As we did not investigate frequency of use or quantity, this should be investigated in the near future. As a consequence, our results reflect the total prevalence of respondents using a product at least once in this period. This may inflate the results for prevalence of use; on the other hand, others have questioned nutritional supplement use in the last 12 months [27], which can be seen as indicative for the (absolute) prevalence of this type of product and the difference in proportion between products and categories.

Since we were limited by the number of questions that could be included in the questionnaire, we chose to pre-select the most relevant types of DS’s and SNPs. In the case of SNPs, sports energy bars are missing in this category because we focused on fluid-based SNPs; therefore, the prevalence of SNP use in our study could be underestimated, but should at least give a representative picture of fluid-based SNP use. It could be debated why, for example, ergogenic supplements such as creatine and B-alanine were classified as DS’s, as others would put these in the SNPs category because of their sports-performance-enhancing claims. On the other hand, some but not all of these ergogenic supplements could be used for health purposes as well. For example, there is a relation between the nitrate in beetroot juice and blood pressure. Quercitin is a polyfenol that could also be related to health and caffeine and seen from a healthy life style perspective, for example, to stimulate energy metabolism to enhance body weight loss. As there is not a single definition, either legal or within nutritional science, of what constitutes a dietary supplement [28], we chose to combine all supplements based on tablets, capsules, drops, and so on in small doses, mostly defined in mg or mcg. As the included sport nutrition products were defined as those delivering substantial energy, macronutrients, fluid, or a combination of the three. In this case, classifying ergogenics as DS’s could affect the prevalence of sport-specific use as a reason. On the other hand, the prevalence of the reason for physical performance regarding DS’s is only 14%, compared with 79% using DS’s for health purposes. The contribution of these ergogenic supplements on sport-specific use can be stated as limited. Still, this can be seen as a limitation that could have been prevented if we divided these categories beforehand.

As health is the main reason for use of DS’s, SNPs, or both, these observations suggest that the general population has the impression that both DS’s and SNPs are considered a healthy choice. An obvious question is whether the majority of this population needs these products, and whether the products used actually enhanced their sporting performance or influenced their health.

## 5. Conclusions

This study shows a high prevalence of DS and SNP use among the general population. Dietary supplement use was most frequently reported by women and older people, while men, young people, and those exercising most frequently reported the highest use of SNPs. Next to improving health, improvement of physical performance appears to be an important objective for both DS’s and SNPs, and the combined use of these products. It can be questioned whether the use of SNPs fits all respondents’ physical activity needs and in particular for those classified as being sedentary.

## Figures and Tables

**Table 1:** Prevalence of use of dietary supplements and sports nutrition products.

		Gender		Age (Years)					Sporting Frequency per Year				
	Total	Men	Women	15–20	21–35	36–50	51–65	66–80	0	1–11	12–59	60–119	>120
		(*n* = 791)	(*n* = 753)	(*n* = 111)	(*n* = 358)	(*n* = 400)	(*n* = 422)	(*n* = 253)	(*n* = 401)	(*n* = 137)	(*n* = 484)	(*n* = 275)	(*n* = 247)
		A	B	C	D	E	F	G	H	I	J	K	L
**Dietary supplements**													
Multivitamins/minerals	28 (26–30)	23 (20–26)	32 (29–35) a	26 (18–34)	32 (27–37) f,g	29 (25–31)	25 (21–29)	23 (18–28)	22 (18–26)	24 (17–31)	27 (23–31)	34 (28–40) h,i,j	34 (28–40) h,i,j
Vitamin C	19 (17–21)	17 (14–20)	21 (18–24)	20 (13–27)	23 (19–27) g	19 (15–23)	19 (15–23)	13 (9–17)	15 (11–19)	17 (11–23)	20 (16–24)	22 (17–27) h	22 (17–27) h
Vitamin D	16 (14–18)	12 (10–14)	20 (17–23) a	13 (7–19)	17 (13–21)	12 (9–15)	16 (12–18)	21 (16–26) e	14 (10–18)	16 (10–22)	15 (12–18)	17 (13–21)	18 (13–23)
Calcium	8 (7–9)	5 (3–7)	11 (9–13) a	7 (2–12)	6 (3–9)	5 (3–7)	11 (8–14) d,e	14 (10–18) c,d,e	7 (5–9)	7 (3–11)	6 (4–8)	9 (5–13)	13 (9–17) h,j
Magnesium	7 (6–8)	3 (2–4)	10 (8–12) a	10 (4–16) d,e	5 (3–7)	4 (2–6)	9 (6–12) d,e	9 (5–13) e	7 (5–9)	5 (1–9)	5 (3–7)	6 (3–9)	11 (7–15) h,j,k
Essential fatty acids/fish oil	5 (4–6)	3 (2–4)	6 (4–8) a	6 (2–10)	4 (2–6)	5 (3–7)	6 (4–8)	3 (1–5)	3 (1–5)	4 (1–7)	6 (4–8)	6 (3–9)	4 (1–7)
Iron	5 (4–6)	4 (3–5)	7 (5–9) a	8 (3–13)	7 (4–10)	5 (3–7)	4 (2–6)	5 (2–8)	4 (2–6)	6 (2–10)	5 (3–7)	7 (4–10)	8 (5–11) h
Caffeine	5 (4–6)	6 (4–8)	5 (4–6)	16 (9–23) d,e,f,g	8 (5–11) e,f,g	3 (1–5)	3 (1–5)	2 (0–4)	3 (1–5)	3 (0–6)	6 (4–8) h	6 (3–9) h	8 (5–11) h
Probiotics	7 (6–8)	4 (3–5)	9 (7–11) a	7 (2–12)	7 (4–10)	7 (5–9)	7 (5–9)	5 (2–8)	5 (3–7)	5 (2–8)	7 (5–9)	8 (5–11)	7 (4–10)
Zinc	3 (2–4)	3 (2–4)	2 (1–3)	2 (0–4)	3 (1–5)	2 (0–4)	3 (1–5)	3 (1–5)	1 (0–2)	4 (1–7)	3 (1–5)	2 (0–4)	4 (1–7) h
Creatine	1 (0–2)	1 (0–2)	1 (0–2)	1 (−1–3)	2 (1–3) f	1 (0–2) f	0	1 (1–3)	0	0	1 (0–2)	0	4 (1–7) h,j
B-alanine	1 (0–2)	1 (0–2)	1 (0–2)	1 (−1–3)	1 (0–2)	0	1 (0–2)	1 (0–2)	0	1 (0–2)	1 (0–2)	0	2 (0–4) h
Beetroot juice/nitrate	1 (0–2)	1 (0–2)	1 (0–2)	0	0	2 (1–3)	1 (0–2)	2 (0–4)	0	1 (0–2)	1 (0–2)	2 (0–4)	2 (0–4)
Sodium bicarbonate	1 (0–2)	1 (0–2)	1 (0–2)	1 (−1–3)	0	1 (0–2)	1 (0–2)	1 (0–2)	1 (0–2)	1 (0–2)	0	1 (0–2)	1 (0–2)
HMB *	0	1 (0–2)	0	2 (0–4)	0	1 (0–2)	0	0	0	1 (0–2)	1 (0–2)	0	1 (0–2)
Quercetin	0	0	0	0	0	1 (0–2)	0	0	0	1 (0–2)	0	0	0
Other than these DS’s	4 (3–5)	3 (2–4)	5 (3–7) a	5 (0–10)	6 (3–9) e,f	3 (1–5)	3 (1–5)	3 (1–5)	3 (1–5)	5 (2–8)	3 (2–4)	5 (2–8)	7 (4–10) h,j
None	51 (50–52)	57 (56–58)	44 (42–46)	55 (41–59)	43 (40–46)	53 (51–55)	52 (50–54)	54 (52–56)	59 (57–61)	49 (46–52)	50 (49–51)	47 (44–50)	44 (41–47)
**Sports nutrition products**													
Energy drinks	22 (20–24)	27 (24–30) a	16 (13–19)	43 (34–52) e,f,g	36 (31–41) e,f,g	21 (17–25) f,g	13 (10–16) g	2 (0–4)	10 (7–13)	21 (14–28) h	25 (21–29) h	26 (5) h	29 (6) h
Isotonic drinks	19 (17–21)	23 (20–26) a	15 (12–18)	37 (28–46) d,e,f,g	27 (22–32) e,f,g	21 (17–25) f,g	11 (8–14) g	5 (2–8)	5 (3–7)	17 (11–23) h	20 (16–24) h	28 (5) h,i,j	30 (6) h,i,j
Protein shakes	5 (4–6)	5 (3–7)	5 (3–8)	7 (2–12) f,g	10 (7–13) e,f,g	6 (4–8) f,g	3 (2–4)	2 (0–4)	2(1–3)	3 (0–6)	5 (3–7) h	7 (3) h	14 (4) h,i,j,k
Recovery drinks	3 (2–4)	5 (3–7) a	1 (0–2)	7 (2–12) f	5 (3–7) f	3 (1–5)	1 (−1–3)	0	0	1 (-1–3)	3 (1–5)	4 (2)	8 (3) i,j,k
Energy gels	1 (0–2)	1 (0–2)	1 (0–2)	1 (−1–3)	1 (0–2)	2 (1–3) f	0	0	0	1 (0–2)	0	1 (1)	4 (2) j,k
None of these SNPs	66 (64–68)	61 (58–64)	72 (69–75)	38 (37–39)	49 (48–50)	64 (63–65)	80 (76–84)	92 (88–96)	86 (85–87)	67 (66–68)	64 (60–68)	55 (54–56)	52 (51–53)

Sample size *n* = 1544, and sample size per subgroup was given as (*n*). All data is presented as a percentage and 95 CI. Capital letters are used as a label for subgroups within categories for gender, age, and sporting frequency. Lower case letters displayed below a specific value indicate that this value is significantly larger than the corresponding capital letters. * HMB: beta-hydroxy-beta-methylbutyrate.

**Table 2:** Combined prevalence of use or non-use of dietary supplements and sports nutrition products or both for different groups.

		Age (Years)					Sporting Frequency per Year				
	Total	15–20	21–35	36–50	51–65	66–80	0	1–11	12–59	60–119	>120
		(*n* = 111)	(*n* = 358)	(*n* = 400)	(*n* = 422)	(*n* = 253)	(*n* = 401)	(*n* = 137)	(*n* = 484)	(*n* = 275)	(*n* = 247)
		C	D	E	F	G	H	I	J	K	L
DS’s	28 (26–30)	10 (4–16)	22 (18–26) c	23 (19–27) c	38 (34–42) c,d,e	40 (34–46) c,d,e	35 (30–40) j,k,l	27 (19–35)	27 (24–31)	24 (19–29)	25 (20–30)
SNPs	12 (10–14)	27 (19–35) d,e,f,g	16 (12–20) f,g	13 (10–16) g	9 (6–12) g	2 (0–4)	8 (5–11)	9 (4–14)	12 (9–15) h	16 (12–18) h,i	17 (12–22) h,i
Both	21 (19–23)	35 (26–44) e,f,g	35 (30–40) e,f,g	24 (20–28) f,g	11 (7–15) g	6 (3–9)	7 (5–9)	24 (17–31) h	24 (20–28) h	29 (24–33) h	31 (25–37) h,j
None	38 (36–40)	28 (20–36)	27 (22–32)	41 (36–47) c,d	43 (38–48) c,d	52 (46–58) c,d,e,f	51 (46–56) i,j,k,l	40 (32–48)	37 (33–41)	31 (25–36)	27 (21–33)

Sample size *n* = 1544, and sample size per subgroup was given as (*n*). All data is presented as a percentage and 95 CI. DS = dietary supplement; SNP = sports nutrition product. Capital letters are used as a label for subgroups within categories for gender, age, and sporting frequency. Lower case letters displayed below a specific value indicate that this value is significantly larger than the corresponding capital letters.

**Table 3:** Percentage of health or performance reasons for the use of dietary supplements and sports nutrition products.

		Gender		Age (Years)					Sporting Frequency per Year				
	Total	Men	Women	15–20	21–35	36–50	51–65	66–80	0	1–11	12–59	60–119	>120
		(*n* = 791)	(*n* = 753)	(*n* = 111)	(*n* = 358)	(*n* = 400)	(*n* = 422)	(*n* = 253)	(*n* = 401)	(*n* = 137)	(*n* = 484)	(*n* = 275)	(*n* = 247)
		A	B	C	D	E	F	G	H	I	J	K	L
**Dietary supplements**													
Health	79 (77–81)	72 (69–75)	84 (80–88) a	61 (53–69)	74 (69–79)	81 (76–86) c	85 (80–90) c,d	85 (79–91) c,d	89 (84–94) i,j,k,l	79 (71–87) i	79 (75–83) i	80 (74–86) i	66 (60–72)
Performance	3 (2–4)	5 (4–6) b	1 (0–2)	4 (1–7)	4 (2–6) f	4 (3–5) f	1 (0–2)	1 (0–2)	0	8 (5–11)	3 (2–4)	2 (1–3)	3 (1–5)
Both	11 (10–12)	17 (15–19) b	7 (6–8)	25 (19–31) d,c,f,g	12 (9–15)	9 (7–11)	9 (7–11)	9 (7–11)	1 (0–2)	7 (4–10) h	11 (9–13) h	14 (11–17) h	22 (18–26) h,i,j
Other	7 (6–8)	6 (5–7)	9 (7–11)	10 (6–14)	11 (9–13)	6 (4–8)	6 (4–8)	5 (3–7)	10 (8–12) k	6 (3–9)	8 (6–10)	4 (2–6)	9 (6–12)
**Sports nutrition products**													
Health	29 (27–31)	25 (23–27)	33 (31–35)	17 (11–23)	25 (22–28)	32 (29–35) c	40 (37–43) c,d	43 (41–45) c	31 (29–33) i	35 (30–40) i	29 (26–32) i	33 (29–37) i	18 (14–22)
Performance	12 (11–13)	15 (13–17) b	7 (6–8)	8 (4–12)	16 (13–19) f	12 (10–14) f	4 (3–5)	5 (4–6)	0	9 (6–12)	10 (8–12)	10 (7–13)	23 (19–27) i,j,k
Both	23 (22–24)	28 (26–30) b	17 (15–29)	28 (21–35) d	17 (14–20)	24 (21–27)	29 (27–31) d	29 (27–31)	2 (1–3)	11 (8–14) h	26 (23–29) h,i	23 (19–27) h	34 (29–39) h,i
Other	37 (35–39)	32 (30–34)	43 (41–45) a	46 (38–54) e,f	41 (37–44) f	32 (29–35)	27 (25–29)	24 (22–26)	67 (64–70)	45 (39–51)	34 (31–37)	33 (29–37)	25 (21–29)

Sample size *n* = 1544, and sample size per subgroup was given as (*n*). All data is presented as a percentage and 95 CI. Capital letters are used as a label for subgroups within categories for gender, age and sporting frequency. Lower case letters displayed below a specific value indicate that this value is significantly larger than the corresponding capital letters.

## Abbreviations

The following abbreviations are used in this manuscript:

DS’s	Dietary Supplements
SNPs	Sport Nutrition Products
NSO	Dutch National Sport Study (in Dutch: Nationaal SportOnderzoek)
DSSS	Dutch Sport nutrition and Supplement Study
